# 

**Published:** 1893-12

**Authors:** 


### Society Notes.

**South-East Texas Medical Society.**—The next meeting of this flourishing organization will be held in Beaumont, January 2, 1894. Papers will be read as follows: "On Uterine Hemorrhage," by Dr. D. C. Hewson, of Orange. "Puerperal Eclampsia,'' by Dr. T. B. Selman, of Village Mills; ''Antipyretics in Pneumonia,'' by Dr. J. Saunders, of Orange; ''Plasmodium Malariæ,'' by Dr. J. S. Price, Beaumont; ''Convulsions in Children,'' by Dr. D. M. Childress, of Smith's Tram.; ''Gunshot Wounds of Intestines,'' by Dr. S. W. Sholars, of Orange.

Election of officers will take place at this meeting. Banquet at night under the auspices of the Ladies' Cooking Club.

J. SAUNDERS, M. D., President, Orange, Texas.

---

**Austin District Medical Society.**—The 25th quarterly meeting will be held in Austin, Thursday, the 21st of December. All the respectable physicians accessible to Austin are invited to attend.

The following is the programme:

1. ''Ostitis and Periostitis, with Specimens,'' by Dr. Matthew M. Smith; discussion opened by Dr. Thos. D. Wooten and Dr. J. W. Hamilton.
2. ''A Radical Cure of Varicocele,'' by Dr. S. E. Millican, of New York; discussion opened by Dr. J. W. McLaughlin and Dr. R. S. Gregg.
3. ''Consumption; Shall It Be Quarantined?'' General discussion.
4. Presidential Address by Dr. R. P. Talley.
5. Election of officers.
6. Banquet.

T. J. BENNETT, M. D., Secretary. R. P. TALLEY, M. D., President.

